# Supplementary material for: On the Transition from Control Modes to Spontaneous Modes during ECMO
Source: J Clin Med. 2021 Mar 2;10(5):1001. doi: 10.3390/jcm10051001 (PMC7958116; doi:10.3390/jcm10051001)

SUPPLEMENTARY ONLINE CONTENT

Krista Stephens, Nathan Mitchel, Sean Overton, Joseph E Tonna. On the transition from control modes to spontaneous modes during ECMO. A high-fidelity graphical analysis of ventilatory parameters and changes in pulmonary function among 419 patients.

Supplemental Methods (eMethods)

Figure S1. Patient enrollment flowchart

Supplemental Results (eResults)

Table S1. Ventilator parameters by support mode

Table S2. Subset analysis of ventilator parameters by support mode, *among patients with lung compliance <30 cmH$_2$O*

Table S3. Multivariate analysis of the association between spontaneous mode and tidal volume during the period of transition. (FOR FIGURE 2)

Table S4. Multivariate analysis of the association between spontaneous mode and tidal volume during the period of transition, among patients with lung compliance <30 cmH20.

Table S5. Multivariate analysis of the association between spontaneous mode and PEEP during the period of transition. (FOR FIGURE 1)

Table S6. Multivariate analysis of the association between spontaneous mode and PEEP during the period of transition, among patients with lung compliance <30 cmH2O

Table S7. Multivariate analysis of the association between spontaneous mode and elastic component of mechanical power during the period of transition. (FOR FIGURE 3)

Table S8. Multivariate analysis of the association between spontaneous mode and elastic component of mechanical power during the period of transition, among patients with lung compliance <30 cmH2O.

Table S9. Multivariate analysis of the association between duration of time since transition to spontaneous mode and respiratory rate during the period of transition. (FOR FIGURE S2)

Table S10. Multivariate analysis of the association between duration of time since transition to spontaneous mode and respiratory rate during the period of transition, among patients with lung compliance <30 cmH2O. (FOR FIGURE 4)

Table S11. Multivariate analysis of the association between duration of time since transition to spontaneous mode and PaO2/FiO2 during the period of transition, among patients with tachypnea. (FOR FIGURE 5)

Table S12. Multivariate analysis of the association between duration of time since transition to spontaneous mode and PaO2/FiO2 during the period of transition, among patients without tachypnea. (FOR FIGURE S4)

Table S13. Multivariate analysis of the association between duration of time since transition to spontaneous mode and PaO2/FiO2 during the period of transition, among patients with tachypnea, low compliance subset. (FOR FIGURE S3)

Table S14. Multivariate analysis of the association between duration of time since transition to spontaneous mode and PaO2/FiO2 during the period of transition, among patients without tachypnea, low compliance subset. (FOR FIGURE S5)

Table S15. Multivariate analysis of the association between respiratory rate and PaO2/FiO2 during the period of transition, among patients on control modes. (FOR FIGURE 6)

Table S16. Multivariate analysis of the association between tidal volume and PaO2/FiO2 during the period of transition, among patients on control modes. (FOR FIGURE 6)

Table S17. Multivariate analysis of the association between respiratory rate and PaO2/FiO2 during the period of transition, among patients on control modes, low compliance subset. (FOR FIGURE S6)

Table S18. Multivariate analysis of the association between tidal volume and PaO2/FiO2 during the period of transition, among patients on control modes, low compliance subset. (FOR FIGURE S6)

Figures

Figure S2. Adjusted increase in respiratory rate increases over time since the transition from control mode to spontaneous mode, among all patients

Figure S3. PaO_2/FiO_2 ratio (95% CI) over time among tachypneic patients (respiratory rate ≥30), low compliance subset.

Figure S4. PaO_2/FiO_2 ratio (95% CI) over time among patients without tachypnea (respiratory rate <30).

Figure S5. PaO_2/FiO_2 ratio (95% CI) over time among patients *without* tachypnea (respiratory rate <30), low compliance subset

Figure S6. PaO_2/FiO_2 as a function of tidal volume and respiratory rate among patients with low compliance

Table S1. Ventilator parameters by support mode

Variable[1]	All	Control Modes	Spontaneous Modes	*p*-value[2]
Tidal volume (mL)	468 (360, 584)	428 (310, 534)	513 (413, 633)	<0.0001
Respiratory rate (breaths per minute)	20 (15, 25)	18 (14, 22)	22 (18, 28)	0.32
Peak inspiratory pressure (cmH_2O)	31 (21, 40)	35 (24, 40)	27 (16, 40)	<0.0001
Respiratory system compliance (mL/cmH_2O)	30.3 (22.2, 41)	27.6 (19.4, 37.2)	34.7 (25.6, 43.9)	<0.0001
Positive end expiratory pressure (cmH_2O)	8 (5, 10)	10 (8, 11)	5.4 (5, 8)	<0.0001
Elastic component of mechanical power (mL/(cmH_2O x min)	0.07 (0.04, 0.11)	0.06 (0.03, 0.09)	0.08 (0.05, 0.13)	<0.0001

[1] median, interquartile range (IQR)

Abbreviations: cmH_2O: centimeter of water; mL: milliliter; min: minute

Number of original observations: Tidal volume: 33,940; Respiratory rate: 36,882; Peak inspiratory pressure 33,655; Respiratory system compliance: 10,783; Positive end expiratory pressure: 36,045. Elastic component of mechanical power: 101,949 calculated observations.

[2] *p* value from univariate mixed effects panel regression model of one value per hour, clustered by patient.

Table S2. Subset analysis of ventilator parameters by support mode, among patients with lung compliance <30 cmH_2O

Variable[1]	All	Control Modes	Spontaneous Modes	*p*-value[2]
Tidal volume (mL)	422 (320, 539)	375 (260, 480)	469 (383, 591)	<0.0001
Respiratory rate (breaths per minute)	20 (16, 26)	18 (14, 22)	23 (19, 28)	0.29
Peak inspiratory pressure (cmH_2O)	33 (21, 40)	35 (25, 40)	27 (16, 40)	<0.0001
Respiratory system compliance (mL/cmH_2O)	26.8 (19.1, 36)	23.7 (17, 31)	30.5 (23.4, 41.5)	<0.0001
Positive end expiratory pressure (cmH_2O)	8 (5, 10)	10 (8, 11)	5.5 (5, 8)	<0.0001
Elastic component of mechanical power (mL/(cmH_2O x min)	0.07 (0.04, 0.10)	0.05 (0.02, 0.09)	0.08 (0.05, 0.12)	<0.0001

[1] median, interquartile range (IQR)

Abbreviations: cmH_2O: centimeter of water; mL: milliliter; min: minute

Number of original observations: Tidal volume: 16,514; Respiratory rate: 18,025; Peak inspiratory pressure: 16,454; Respiratory system compliance: 5,496; Positive end expiratory pressure: 17,566. Elastic component of mechanical power: 51,694 calculated observations

[2] *p* value from univariate mixed effects panel regression model of one value per hour, clustered by patient.

Table S3. Multivariate analysis of the association between spontaneous mode and tidal volume during the period of transition

**Variable**	**Coefficient**	**95% CI**	***p*-value**
Spontaneous Mode	59.5	(49.6 to 69.4)	<0.0001
PEEP, *per* cmH_20	−0.1	(−1.7 to 1.3)	0.85
Compliance, *per mL/cmH2O*	3.1	(2.8 to 3.4)	<0.0001
Respiratory rate, *per breath per minute*	−4.8	(−5.3 to −4.3)	<0.0001
Duration of time since switch to spontaneous, *per hour*	1.1	(0.6 to 1.5)	<0.0001

Abbreviations: PEEP: positive end expiratory pressure; mL: milliliters; cmH_2O: centimeter of water;
Multivariate mixed effects panel regression model of one value per hour, clustered by patient.

Table S4. Multivariate analysis of the association between spontaneous mode and tidal volume during the period of transition,
among patients with lung compliance <30 cmH_2O

**Variable**	**Coefficient**	**95% CI**	***p*-value**
Spontaneous Mode	53.9	(40.3 to 67.5)	<0.0001
PEEP, *per* cmH_20	−1.6	(−3.5 to 0.4)	0.12
Compliance, *per mL/cmH2O*	3.3	(2.9 to 3.7)	<0.0001
Respiratory rate, *per breath per minute*	−3.6	(−4.3 to −3.0)	<0.0001
Duration of time since switch to spontaneous, *per hour*	0.9	(0.2 to 1.5)	0.007

Abbreviations: PEEP: positive end expiratory pressure; mL: milliliters; cmH_2O: centimeter of water;
Multivariate mixed effects panel regression model of one value per hour, clustered by patient.

Table S5. Multivariate analysis of the association between spontaneous mode and PEEP during the period of transition

Variable	Coefficient	95% CI	*p*-value
Spontaneous Mode	−0.5	(−0.6 to 0.4)	<0.0001
Tidal volume, *per mL*	0.0	(0.0 to 0.0)	0.78
Compliance, *per mL/cmH2O*	0.0	(0.0 to 0.0)	<0.0001
Respiratory rate, *per breath per minute*	0.1	(0.0 to 0.0)	0.007
Duration of time since switch to spontaneous, *per hour*	0.0	(0.0 to 0.0)	<0.0001

Abbreviations: PEEP: positive end expiratory pressure; mL: milliliters; cmH_2O: centimeter of water;
Multivariate mixed effects panel regression model of one value per hour, clustered by patient.

Table S6. Multivariate analysis of the association between spontaneous mode and PEEP during the period of transition, among patients with lung compliance <30 cmH_2O

Variable	Coefficient	95% CI	*p*-value
Spontaneous Mode	−0.4	(−0.6 to −0.1)	0.02
Tidal volume, *per mL*	0.0	(0.0 to 0.0)	0.09
Compliance, *per mL/cmH2O*	0.0	(0.0 to 0.0)	<0.0001
Respiratory rate, *per breath per minute*	0.0	(0.0 to 0.0)	0.58
Duration of time since switch to spontaneous, *per hour*	0.0	(0.0 to 0.0)	<0.0001

Abbreviations: PEEP: positive end expiratory pressure; mL: milliliters; cmH_2O: centimeter of water;
Multivariate mixed effects panel regression model of one value per hour, clustered by patient.

Table S7. Multivariate analysis of the association between spontaneous mode and elastic component of mechanical power during the period of transition

Variable	Coefficient	95% CI	*p*-value
Spontaneous Mode	0.0	(0.0 to 0.0)	<0.0001
PEEP, *per cmH₂0*	0.0	(0.0 to 0.0)	0.1
Compliance, *per mL/cmH2O*	0.0	(0.0 to 0.0)	<0.0001
Respiratory rate, *per breath per minute*	0.0	(0.0 to 0.0)	<0.0001
Duration of time since switch to spontaneous, *per hour*	0.0	(0.0 to 0.0)	<0.0001

Abbreviations: PEEP: positive end expiratory pressure; mL: milliliters; cmH_2O: centimeter of water;
Multivariate mixed effects panel regression model of one value per hour, clustered by patient.

Table S8. Multivariate analysis of the association between spontaneous mode and elastic component of mechanical power during the period of transition, among patients with lung compliance <30 cmH_2O

Variable	Coefficient	95% CI	*p*-value
Spontaneous Mode	0.0	(0.0 to 0.0)	<0.0001
PEEP, *per cmH_20*	0.0	(0.0 to 0.0)	0.72
Compliance, *per mL/cmH2O*	0.0	(0.0 to 0.0)	<0.0001
Respiratory rate, *per breath per minute*	0.0	(0.0 to 0.0)	<0.0001
Duration of time since switch to spontaneous, *per hour*	0.0	(0.0 to 0.0)	0.003

Abbreviations: PEEP: positive end expiratory pressure; mL: milliliters; cmH_2O: centimeter of water;
Multivariate mixed effects panel regression model of one value per hour, clustered by patient.

Table S9. Multivariate analysis of the association between duration of time since transition to spontaneous mode and respiratory rate during the period of transition

Variable	Coefficient	95% CI	*p*-value
Duration of time since switch to spontaneous, *per hour*	0.1	(0.07 to 0.14)	<0.0001
PaO_2/FiO_2	0.0	(0.0 to 0.0)	<0.0001
Compliance, *per mL/cmH2O*	0.0	(−0.1 to −0.06)	<0.0001
PEEP, *per* cmH_20	0.16	(0.02 to 0.0)	0.02

Abbreviations: PEEP: positive end expiratory pressure; mL: milliliters; cmH_2O: centimeter of water;
Multivariate mixed effects panel regression model of one value per hour, clustered by patient.

Table S10. Multivariate analysis of the association between duration of time since transition to spontaneous mode and respiratory rate during the period of transition, among patients with lung compliance <30 cmH_2O

Variable	Coefficient	95% CI	*p*-value
Duration of time since switch to spontaneous, *per hour*	0.14	(0.1 to 0.2)	<0.0001
PaO_2/FiO_2	0.0	(0.0 to 0.0)	0.03
Compliance, *per mL/cmH2O*	−0.1	(−0.1 to −0.04)	<0.0001
PEEP, *per* cmH_20	0.14	(−0.07 to 0.35)	0.2

Abbreviations: PEEP: positive end expiratory pressure; mL: milliliters; cmH_2O: centimeter of water;
Multivariate mixed effects panel regression model of one value per hour, clustered by patient.

Table S11. Multivariate analysis of the association between duration of time since transition to spontaneous mode and PaO2/FiO2 during the period of transition, among patients with tachypnea

**Variable**	**Coefficient**	**95% CI**	***p*-value**
Duration of time since switch to spontaneous, *per hour*	−1.9	(−3.1 to −0.7)	0.001
Compliance, *per mL/cmH2O*	0.1	(−1 to 1)	0.89
PEEP, *per cmH$_2$0*	−8.0	(−12.5 to −3.1)	0.001
Respiratory rate, *per breath per minute*	−3.7	(−5.7 to −1.7)	<0.0001

Abbreviations: PEEP: positive end expiratory pressure; mL: milliliters; cmH$_2$O: centimeter of water;

Tachypnea defined as respiratory rate ≥30.

Multivariate mixed effects panel regression model of one value per hour, clustered by patient.

Table S12. Multivariate analysis of the association between duration of time since transition to spontaneous mode and PaO2/FiO2 during the period of transition, among patients without tachypnea

**Variable**	**Coefficient**	**95% CI**	***p*-value**
Duration of time since switch to spontaneous, *per hour*	0.2	(−0.3 to 0.7)	0.41
Compliance, *per mL/cmH2O*	1.3	(0.9 to 1.7)	<0.0001
PEEP, *per* cmH_20	0.1	(−2.1 to 2.2)	0.95
Respiratory rate, *per breath per minute*	−1.2	(−2.0 to 0.4)	0.002

Abbreviations: PEEP: positive end expiratory pressure; mL: milliliters; cmH_2O: centimeter of water;

Tachypnea defined as respiratory rate ≥30.

Multivariate mixed effects panel regression model of one value per hour, clustered by patient.

Table S13. Multivariate analysis of the association between duration of time since transition to spontaneous mode and PaO2/FiO2 during the period of transition, among patients with tachypnea, low compliance subset

**Variable**	**Coefficient**	**95% CI**	***p*-value**
Duration of time since switch to spontaneous, *per hour*	−1.7	(−3.3 to −0.1)	0.036
Compliance, *per mL/cmH2O*	0.19	(−1.2 to 1.5)	0.89
PEEP, *per* cmH_20	−10.6	(−16.3 to −4.9)	<0.0001
Respiratory rate, *per breath per minute*	−1.6	(−4.1 to 0.8)	0.196

Abbreviations: PEEP: positive end expiratory pressure; mL: milliliters; cmH_2O: centimeter of water;

Tachypnea defined as respiratory rate ≥30.

Multivariate mixed effects panel regression model of one value per hour, clustered by patient.

Table S14. Multivariate analysis of the association between duration of time since transition to spontaneous mode and PaO2/FiO2 during the period of transition, among patients without tachypnea, low compliance subset

Variable	Coefficient	95% CI	*p*-value
Duration of time since switch to spontaneous, *per hour*	0.9	(0.14 to 1.6)	0.019
Compliance, *per mL/cmH2O*	1.3	(0.65 to 1.9)	<0.0001
PEEP, *per* cmH_20	5.5.6	(2.2 to 8.7)	0.001
Respiratory rate, *per breath per minute*	−0.9	(−2.2 to 0.3)	0.125

Abbreviations: PEEP: positive end expiratory pressure; mL: milliliters; cmH_2O: centimeter of water;

Tachypnea defined as respiratory rate ≥30.

Multivariate mixed effects panel regression model of one value per hour, clustered by patient.

Table S15. Multivariate analysis of the association between respiratory rate and PaO2/FiO2 during the period of transition, among patients on control modes

**Variable**	**Coefficient**	**95% CI**	***p*-value**
Respiratory rate, *per breath per minute*	−5.1	(−7.6 to −2.5)	<0.0001
Duration of time since switch to spontaneous, *per hour*	−10.5	(−24.9 to 3.9)	0.15
Compliance, *per mL/cmH2O*	0.26	(−0.85 to 1.4)	0.64
PEEP, *per* cmH_20	−2.8	(−8.8 to 3.1)	0.34

Abbreviations: PEEP: positive end expiratory pressure; mL: milliliters; cmH_2O: centimeter of water;
Multivariate mixed effects panel regression model of one value per hour, clustered by patient.

Table S16. Multivariate analysis of the association between tidal volume and PaO2/FiO2 during the period of transition, among patients on control modes

Variable	Coefficient	95% CI	*p*-value
Tidal volume, *per mL*	−0.17	(−0.29 to −0.04)	0.007
Duration of time since switch to spontaneous, *per hour*	−1.8	(−17.6 to 13.8)	0.82
Compliance, *per mL/cmH2O*	1.8	(−0.4 to 3.1)	0.01
PEEP, *per* cmH_20	−1.5	(−7.6 to 4.6)	0.63

Abbreviations: PEEP: positive end expiratory pressure; mL: milliliters; cmH_2O: centimeter of water; Multivariate mixed effects panel regression model of one value per hour, clustered by patient.

Table S17. Multivariate analysis of the association between respiratory rate and PaO2/FiO2 during the period of transition, among patients on control modes, low compliance subset

Variable	Coefficient	95% CI	*p*-value
Respiratory rate, *per breath per minute*	−9.2	(−13.1 to −5.5)	<0.0001
Duration of time since switch to spontaneous, *per hour*	−23.6	(−46.3 to −0.9)	0.041
Compliance, *per mL/cmH2O*	0.08	(−1.57 to 1.7)	0.92
PEEP, *per cmH20*	1.0	(−8.3 to 10.4)	0.83

Abbreviations: PEEP: positive end expiratory pressure; mL: milliliters; cmH_2O: centimeter of water;
Multivariate mixed effects panel regression model of one value per hour, clustered by patient.

Table S18. Multivariate analysis of the association between tidal volume and PaO2/FiO2 during the period of transition, among patients on control modes, low compliance subset

Variable	Coefficient	95% CI	*p*-value
Tidal volume, *per mL*	−0.3	(−0.49 to −0.11)	0.002
Duration of time since switch to spontaneous, *per hour*	−10.6	(−34.6 to 13.3)	0.38
Compliance, *per mL/cmH2O*	2.8	(0.5 to 5.1)	0.013
PEEP, *per* cmH_20	−0.69	(−13.1 to 6.2)	0.49

Abbreviations: PEEP: positive end expiratory pressure; mL: milliliters; cmH_2O: centimeter of water;
Multivariate mixed effects panel regression model of one value per hour, clustered by patient.

Figure S1. Patient enrollment flowchart

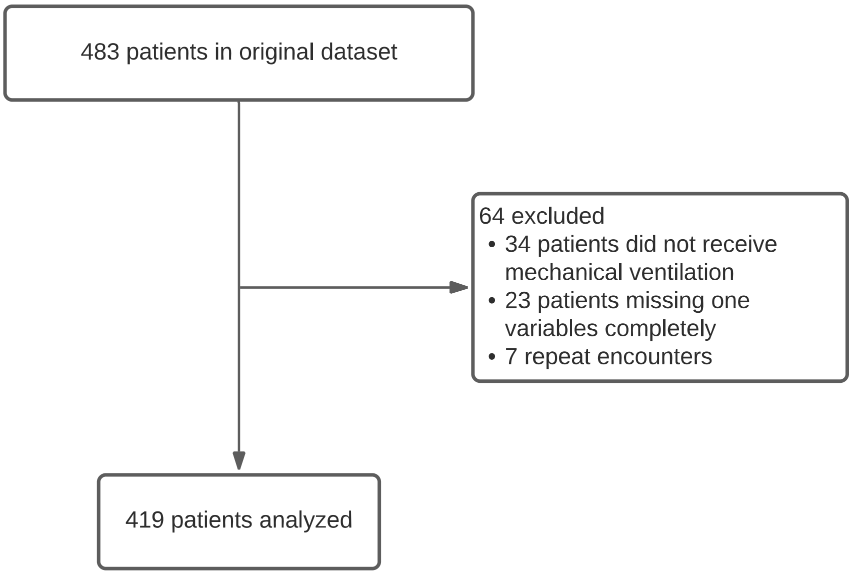

Figure S2. Adjusted increase in respiratory rate increases over time since the transition from control mode to spontaneous mode, among patients

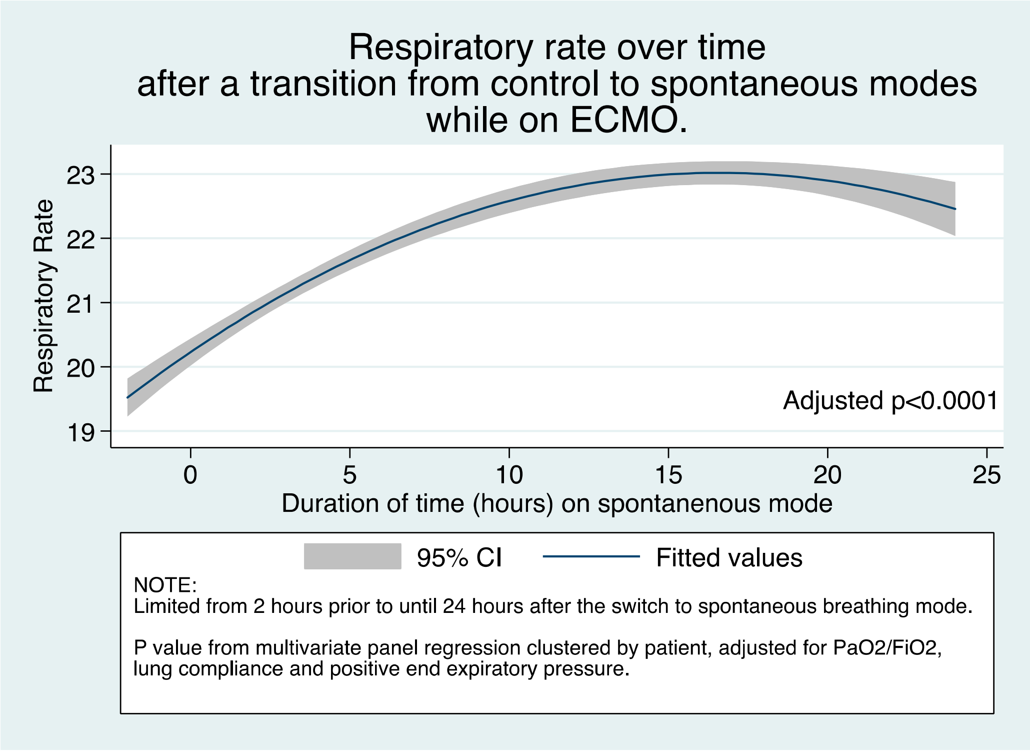

Figure S3. PaO_2/FiO_2 ratio (95% CI) over time among tachypneic patients (respiratory rate ≥30), low compliance subset.

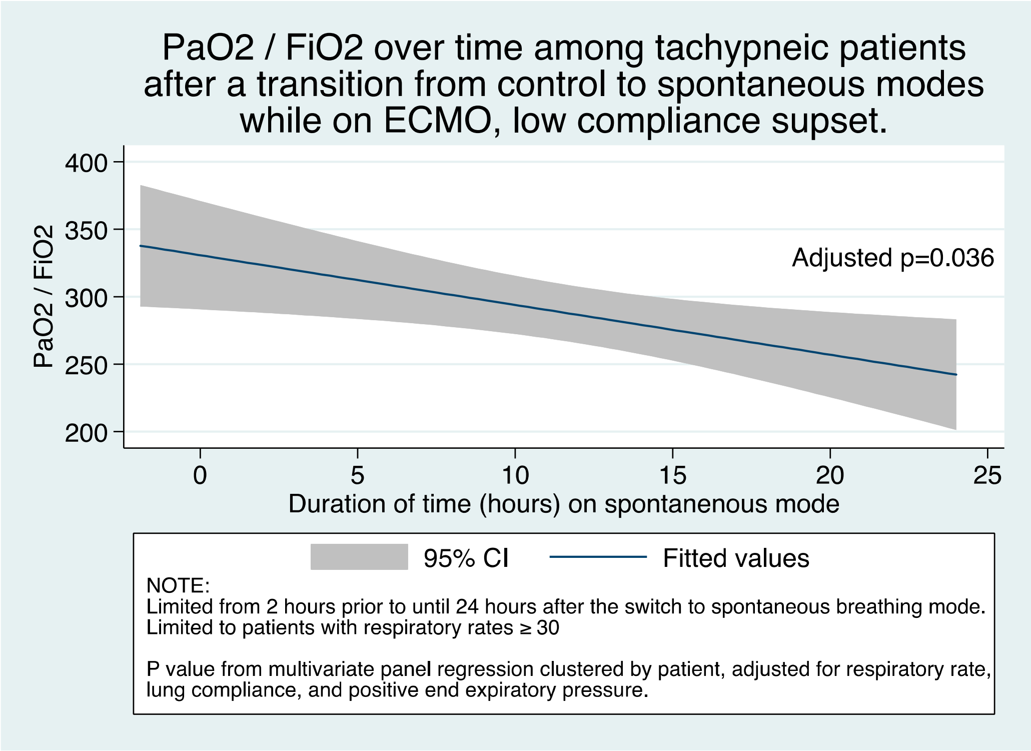

Figure S4. PaO_2/FiO_2 ratio (95% CI) over time among patients without tachypnea (respiratory rate <30).

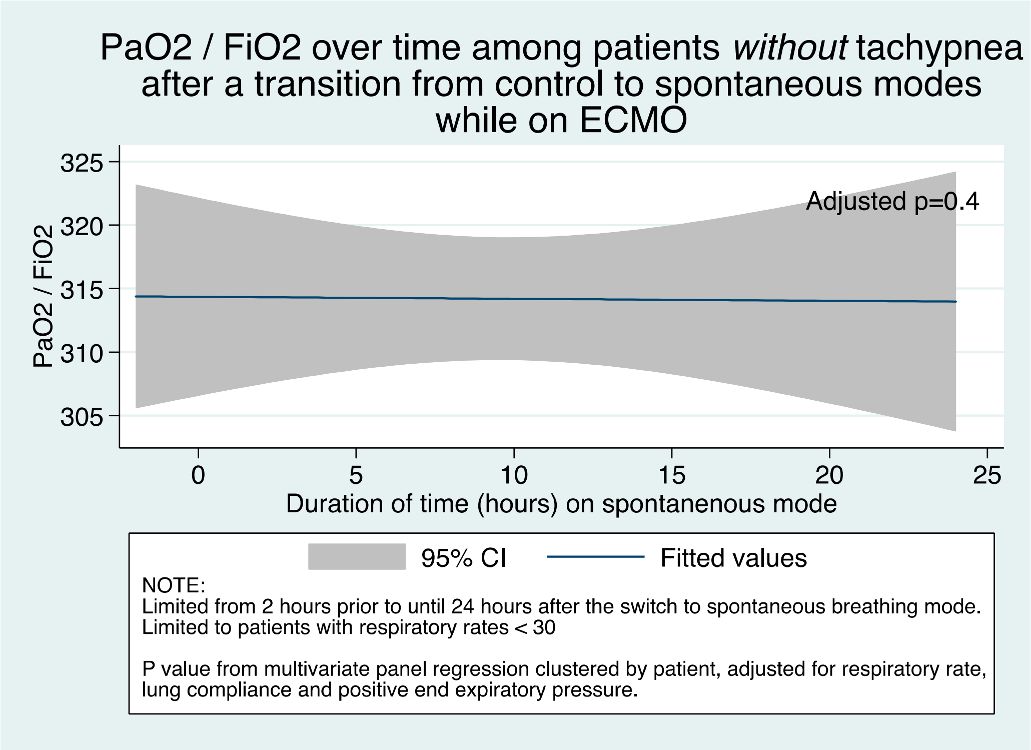

Figure S5. PaO_2/FiO_2 ratio (95% CI) over time among patients *without* tachypnea (respiratory rate <30), low compliance subset

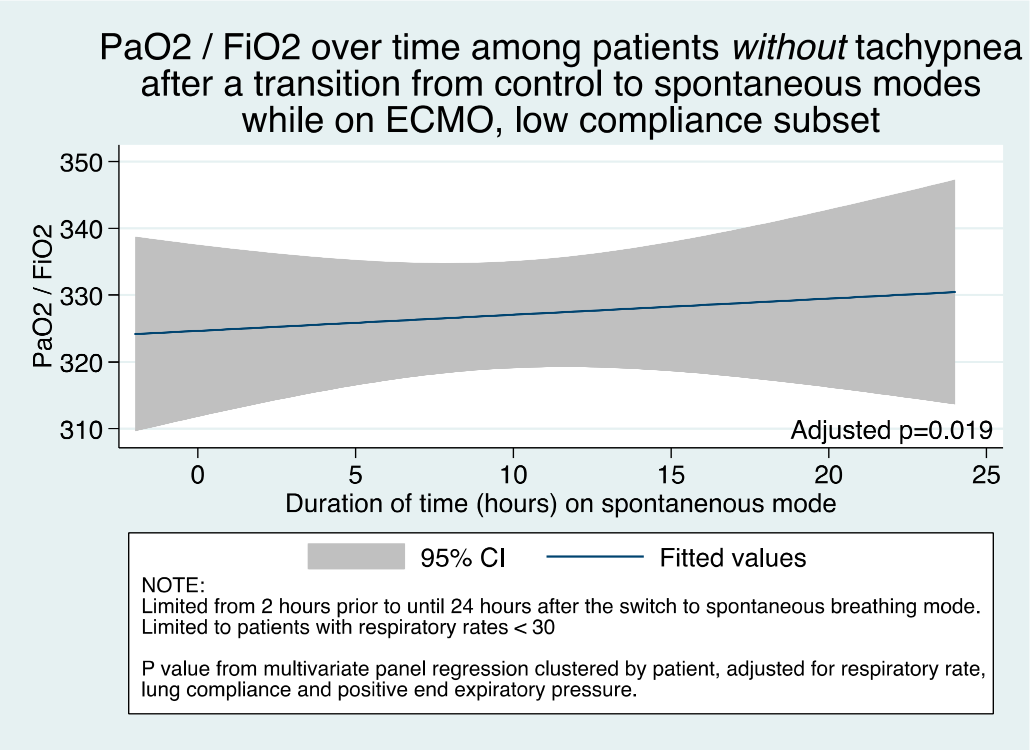

Figure S6. PaO_2/FiO_2 as a function of tidal volume and respiratory rate among patients with low compliance

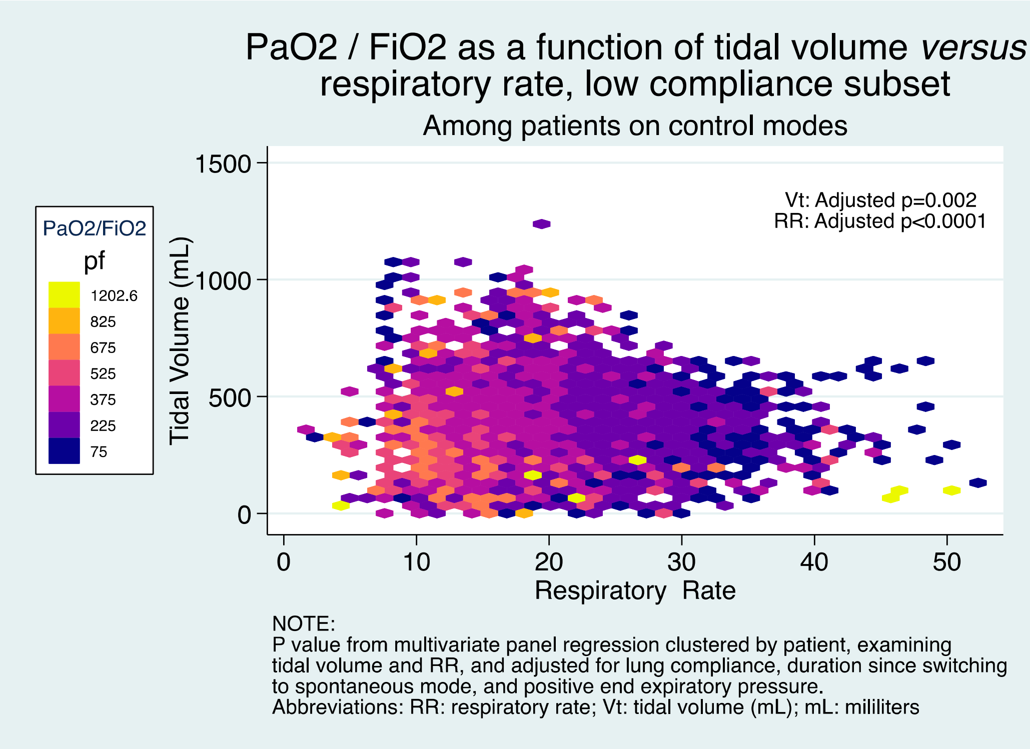

Supplement: Supplementary file 1 [file jcm-10-01001-s001.pdf]
